# Deciphering heat wave effects on wheat grain: focusing on the starch fraction

**DOI:** 10.3389/fpls.2024.1459283

**Published:** 2024-12-04

**Authors:** Rita Pereira, Diana Tomás, Manuela Silva

**Affiliations:** ^1^ Instituto Superior de Agronomia, Universidade de Lisboa, Tapada da Ajuda, Lisboa, Portugal; ^2^ LEAF—Linking Landscape, Environment, Agriculture and Food Research Center, Instituto Superior de Agronomia, Universidade de Lisboa, Tapada da Ajuda, Lisboa, Portugal; ^3^ Associate Laboratory TERRA, Instituto Superior de Agronomia, Universidade de Lisboa, Tapada da Ajuda, Lisboa, Portugal

**Keywords:** bread wheat, high temperature, starch granules, Lugol iodine, endosperm ultrastructure, SEM, transcription, RNA sequencing

## Abstract

Wheat is an essential staple food, and its production and grain quality are affected by extreme temperature events. These effects are even more relevant considering the increasing food demand for a growing world population and the predicted augmented frequency of heat waves. This study investigated the impact of simulated heat wave (HW) conditions imposed during grain filling on starch granule characteristics, endosperm ultrastructure, and transcriptomic modulation of genes involved in starch synthesis and degradation. All these evaluations were performed with four different genotypes, two commercial wheat varieties (Antequera and Bancal), and two traditional landraces (Ardito and Magueija). Starch granule size distribution and shape were significantly altered by HW treatment, revealing an increase of A-type granules in Ardito and an opposite effect in Magueija and Bancal, while Antequera remained stable. Analysis of the largest (LD) and smallest (SD) granule diameters also revealed genotype-specific changes, with Magueija showing a shift toward more spherical A-type granules after the HW treatment. Scanning electron microscopy confirmed alterations in endosperm morphology, including increased vitreousness in Bancal and substantial increase of endosperm cavities and grain size reduction in Magueija under HW stress. The transcriptomic analysis confirmed the stability of Antequera under HW, in contrast with the other genotypes where differential gene expression related to starch metabolism was detected. These effects were particularly severe in Magueija with the downregulation of genes encoding for enzymes involved in amylopectin synthesis (both starch synthases and starch-branching enzyme) and upregulation of α-amylase-encoding genes. These findings contribute to the understanding of heat stress effects on wheat grain quality, emphasize the importance of genetic diversity in HW responses, and suggest potential avenues for breeding climate-resilient wheat varieties.

## 1 Introduction

Wheat is the third most-produced cereal in the world with an annual production of 810 × 10^6^ t and represents a major global food supply equivalent to 1.5 × 10^9^ kcal according to the latest Food and Agriculture Organization Corporate Statistical Database (FAOSTAT) Food Balance for 2022 (Food and Agriculture Organization Statistics [FAOSTAT], 2022). Wheat grain comprises three components: the embryo (germ), the endosperm, and the outer layers (bran). The most abundant is the endosperm (80%–85%), which consists of the outer aleurone layer and the starchy endosperm, where starch accumulates within an extensive proteinaceous matrix (reviewed in Zhou et al., 2018). From the wheat whole grain composition, starch stands out, reaching 65%–75% (Shewry, 2009), contributing to its nutritional value and influencing the technological properties of wheat final products (Maningat and Seib, 2010). This polysaccharide is essentially composed of two different glucose polymers—amylose (~20% to 30%) and amylopectin (~70% to 80%)—and its biosynthesis and storage occur in amyloplasts forming insoluble particles called reserve granules (Shewry, 2009). In addition to being actively involved in carbohydrate metabolism, amyloplasts have also been implicated in other functions, namely, stress response, defense, and transport processes, affecting grain characteristics with an impact on yield and quality (Ma et al., 2018). Starch granules are classified into two categories: A-type starch granules, larger than 10 µm in diameter, presenting a lenticular shape and containing more amylose, and their biosynthesis is more precocious, beginning 4 days after anthesis (DAA); B-type starch granules, which are smaller than 10 µm in diameter and predominantly spherical, start to develop 10–12 DAA (Shewry et al., 2020; Ma et al., 2018). Amylose and amylopectin are two glucan polymers that differ in their structure: amylose, consisting of predominantly linear chains of α1–4 glycosidic linkages, and amylopectin, which has a branched structure with both α1–4 and α1–6 glycosidic linkages (Hurkman et al., 2003). The first step in starch synthesis is the conversion of glucose-1-phosphate in ADP-glucose by plastid-located ADP-glucose pyrophosphorylase (AGPase; EC 2.7.7.27), the substrate for starch synthases (SS; EC 2.4.1.21). Six starch synthase isoforms have been identified to date: SSI, SSII, SSIII, and SSIV, which are involved in amylopectin synthesis, each with a specific role in starch biosynthesis; and two granule-bound starch synthases (GBSS), GBSSI and GBSSII, mainly involved in amylose synthesis in storage and non-storage tissues, respectively. Starch-branching enzyme (SBE; EC 2.4.1.18) introduces new branches on starch molecules, mainly amylopectin, by cleaving the internal α-(1→4) glycosidic linkage of a branch chain and creating a new α-(1→6) glycosidic linkage (reviewed in Wang et al., 2014; Zhang et al., 2015).

The Earth’s climate continues to evolve; it is clear that global warming is causing a range of adverse effects that may ultimately lead to an imbalance in food security (IPCC, 2023). Therefore, it is imperative to understand how thermal stress affects major crops such as wheat. Wheat is currently produced across the globe in different environmental conditions, and its yield and quality are affected by several environmental factors, including temperature. An editorial published in 2023 on *Frontiers in Plant Science* (Aloisi et al., 2023) pointed out that the implications of climate change on wheat production are particularly severe, which is more worrying given the need to expand its production to meet increasing global demand. Indeed, Guo et al. (2024), anticipating the consequences of 1.5°C and 2°C warming targets on the global sustainable distribution of wheat, suggested an increase in the supply–demand disparity. Some of the main effects of temperatures higher than the optimum (12°C to 22°C; Porter and Gawith, 1999) imposed during wheat grain development have long been studied, reporting the decrease in the number of grains per spike, grain shrinkage, grain development shortage, decrease of starch synthesis, and increase in the grain-filling rate (Farooq et al., 2011). Altogether, it has been estimated that for each degree Celsius above the ideal temperature for anthesis and grain filling, amylose and amylopectin syntheses are strongly affected, reducing wheat production by approximately 4%–6% (Liu et al., 2016). However, recent field studies evaluating a single wheat cultivar suggest that a warmer growing season may result in higher grain yield and protein content (Yu et al., 2023). The assessment of the performance of the European variety Axona under daily high temperatures from anthesis through grain maturation revealed a decrease in starch content at harvest associated with reduced activities of the enzyme involved in endosperm starch deposition (Harris et al., 2023). The controlled imposition of 4 days of heat treatment reaching 35°C during the day revealed that earlier treatments after anthesis induce loss of grain yield, while later treatments induce a decrease in starch quality (Liu et al., 2023). The effect of a 0.32°C post-anthesis temperature increment resulting from late sowing was estimated to induce a 1.2% decrease in total starch content (Zhang et al., 2023a). However, Kino et al. (2020) conducted an analysis through RNA sequencing of grains subjected to post-anthesis high-temperature treatment and found a significant downregulation of pericarp genes that regulate cell wall expansion, which may be related to endosperm growth restriction. These reports suggest that the plasticity of wheat plants in a changing climate may be complex, depending on the wheat genotype considered and the particular thermal condition studied. RNA sequencing of developing grains from Australian genebank genotypes subjected to heat stress 3 days after anthesis identified different sets of responsive genes in tolerant vs. susceptible genotypes (Rangan et al., 2020). Genotypic variability in heat response was also revealed by studying different commercial and traditional bread wheat varieties submitted to heat wave-like treatments (Tomás et al., 2020a; b; c). Heat waves are periods of five or more consecutive days with a daily maximum temperature of at least 5°C higher than the average maximum temperature, as defined by the World Meteorological Organization (World Meteorological Organization [WMO], 2023). In these works (Tomás et al., 2020a; b; c), different parameters related to grain yield and quality were studied in the same commercial and traditional varieties of bread wheat evaluated in this report. Several significant effects were observed, such as a decrease in ten-grain weight in landrace plants treated with heat (Tomás et al., 2020c) as well as a decrease in protein content in the commercial variety Antequera and the opposite effect in Ardito (Tomás et al., 2020b). Furthermore, the transcriptional levels of puroindolines (puroindoline a and puroindoline b) and the relative protein composition (globulin, glutenin, and gliadin) of commercial varieties were also assessed, for which only significant differences were observed in Antequera, showing a globulin and glutenin increase and a decrease in the gliadin/glutenin ratio in grains from treated plants (Tomás et al., 2020a). Evaluation by RT-qPCR of transcription levels of genes encoding high-molecular-weight glutenins, granule‐bound starch synthase, and puroindolines revealed a high intervarietal diversity, although no significant variability was induced by heat wave-like treatments (Tomás et al., 2020a). Whole transcriptome profiles of developing wheat grains of commercial varieties and landraces subjected to heat wave-like treatments during grain filling (Tomás et al., 2022) showed that old traditional varieties had significantly more differentially expressed genes (DEGs) in comparison with the commercial ones. Moreover, commercial varieties of major DEGs were associated with RNA and protein synthesis and metabolic changes, whereas in landraces, upregulated genes encoded heat-responsive proteins such as heat shock proteins. In addition, a NAC transcription factor, which negatively regulates starch synthesis and grain yield, was upregulated in most of the genotypes studied (Tomás et al., 2022).

Since the latest IPCC report reinforces that the frequency and intensity of hot extremes such as heat waves will continue to worsen worldwide (IPCC, 2023), this work aims to discern more deeply the effects of heat waves during grain filling, particularly on the starch fraction, evaluating its effects on starch granule conformation, endosperm ultrastructure, and transcription patterns of starch synthesis and degradation-related genes in different commercial and traditional varieties, contributing to the characterization of relevant bread wheat diversity.

## 2 Materials and methods

### 2.1 Plant material and heat wave treatment

In this study, four genotypes of bread wheat (*Triticum aestivum* L.) were analyzed: two commercial varieties, Bancal (NLI/AGR/ES/TRITI_AES/227602) and Antequera (NLI/AGR/ES/TRITI_AES/227284), and two old Portuguese landraces, Ardito and Magueija (Vasconcelos, 1933). The selection of the genotypes studied resulted from previous works published by our research team (Tomás et al., 2020a, b, c). Seeds of commercial varieties were provided by ANSEME (Associação Nacional dos Produtores e Comerciantes de Sementes, Portugal), and seeds of landraces were obtained from the EAN Germplasm Bank (PRT005, Oeiras, Portugal). The seeds used in this work were obtained after 2 years of controlled propagation to discard any performance differences resulting from epigenetic memory phenomena and were germinated and maintained in growth chambers with 8 hours of darkness at 20°C and 16 hours of light at 25°C for 3 weeks. The plants were then transferred to soil pots and grown in a field greenhouse. When the plants reached anthesis (first anther visualization in the first spike), they were returned to the growth chamber with the above-mentioned conditions, and after 10 days, 10 plants remained in these control conditions, and another 10 were subjected to a high-temperature regime mimicking a heat wave (HW) with a daily maximum temperature of 40°C during 4 hours for 1 week. At the endpoint of the maximum temperature period of the seventh treatment day, two immature grains were collected from the middle of the first spike of each plant grown in control conditions and submitted to the HW treatment. The collected immature grains were stored at −80°C for subsequent RNA extraction. After the treatment period, plants were maintained in the field greenhouse until the end of the life cycle, and all mature grains were collected individually from the first spike of each plant and stored at −20°C. It must be emphasized that both immature grain (for RNA sequencing) and mature grain analyses were performed exclusively in material collected from the first spike of each biological replicate to guarantee identical developmental stages during HW treatments. A schematic representation of the whole assay detail is presented in **Supplementary Figure 1**.

### 2.2 Starch granule evaluation

For starch granule quantification, mature grains of each variety/condition were cut transversely to separate the embryo region from the endosperm. The endosperm regions obtained were individually ground using Cryomill (Retsch GmbH, Haan, Germany). The resulting flour was used to make spreads in 45% acetic acid, and after preparation quality assessment, coverslips were removed using carbon dioxide. Air-dried spreads were stained with 30 μL of a 1:10 Lugol’s dilution (1.0 g of bi-sublimated iodine, 2.0 g of potassium iodide, and 300.0 mL of water) at room temperature for 10 minutes. After washing with distilled water and air-drying, the spreads were mounted in immersion oil and observed using an epifluorescence microscope (Leitz Biomed, Wetzlar, Germany), and photographed using the ZEISS Axiocam image acquisition system. For each genotype/condition, at least two wheat flour spreads (technical replicates) of grains from three different plants (biological replicates) were analyzed.

ImageJ was used to measure the largest and smallest diameters of the starch granules. Image quantification was conducted in raw images by manual selection of the linear measures to obtain. The ratio between the largest and smallest diameters of each granule was calculated afterward to infer the shape of the granules. Mean values and standard deviation were determined for the largest and smallest diameters and their ratio for each variety/condition. A similar number of granules of each genotype/condition were quantified and classified according to their largest diameter: A-type between 10 and 35 µm and B-type <10 µm (Shewry et al., 2020). The mean values obtained were compared using Student’s t-test, and the frequency distribution of starch granule types was calculated and compared using the chi-squared test. Significant differences were considered at p-value <0.05.

### 2.3 Endosperm analysis through scanning electron microscopy

Evaluation of endosperm ultrastructure was performed using scanning electron microscopy (SEM). The endosperm regions of mature grains of each variety/condition, transversely cut as described, were placed in a desiccator for 24 hours prior to SEM observation. Endosperm cross sections were analyzed using the SEM HITACHI TM3030Plus tabletop microscope at different magnifications using energy-dispersive X-ray spectroscopy (EDX) and Mix (mixing image) specifications. This analysis aimed to evaluate the shape and texture of the wheat grains as well as detailed structural aspects of the endosperm starch granules. For each genotype/condition, grains from three different plants (biological replicates) were analyzed, and the figures presented are raw SEM images without any digital processing.

### 2.4 RNA sequencing and differential gene expression analysis

Total RNA was extracted from individual immature grains using the Spectrum™ Plant Total RNA Kit (Sigma-Aldrich, Inc., Madrid, Spain) to obtain three biological replicates per genotype/condition, each consisting of a pool of RNA extracted from three immature grains obtained from three different plants. RNA library preparation and sequencing were performed by the Genomics Unit of the Instituto Gulbenkian Ciência (Oeiras, Portugal) as described in detail in Tomás et al. (2022). Libraries were prepared using the SMART-Seq2 and Nextera protocol, and sequencing was performed on the NextSeq500 Illumina^®^ sequencer using the 75 SE high-throughput kit. Raw reads were trimmed to the longest continuous segment of Phred quality (threshold of 30 or higher) to improve overall base quality and remove adaptors, and trimmed reads were mapped to the *T. aestivum* genome (ftp://ftp.ensemblgenomes.org/pub/plants/release-48/fasta/triticum_aestivum/dna/Triticum_aestivum.IWGSC.dna.toplevel.fa.gz) using hisat2 with default parameters.

Read mapping to genomic features and gene expression quantification were performed using featureCounts, control vs. HW differential gene expression was tested using DESeq2, and a search of gene ID and encoding products was made in Ensemble Plants BioMart. The R software was used to obtain hierarchical clustering of samples for all varieties and conditions showing the relationships between the list of differentially expressed genes of all varieties and conditions. Gene Ontology enrichment analysis was conducted using the AgriGOv22 web-based tool with the following parameter settings: Fisher’s test with Bonferroni multi-test adjustment method, 0.05 significance level, five minimum mapping entries, and complete Gene Ontology. The GO database3 was used to analyze GO term enrichment of DEGs, and the Kyoto Encyclopedia of Genes and Genomes (KEGG) database was used to identify the enriched metabolic pathways, as well as the enzymes involved. The RNA sequencing data analysis pipeline, including all software versions and key parameters used, is further detailed in our previous article (Tomás et al., 2022). The expression levels of genes assessed by the whole transcriptome analysis were validated by RT-qPCR assays performed on the exact same RNA samples for several genes, namely, genes encoding for enzymes involved in starch synthesis, using four endogenous reference genes with stable expression across a wide range of developmental and environmental conditions: *ADP‐ribosylation factor*, *ubiquinol‐cytochrome C reductase iron‐sulfur subunit*, *superoxide dismutase [Cu‐Zn]*, and *glyceraldehyde 3‐phosphate dehydrogenase* (Tomás et al., 2020a).

## 3 Results and discussion

### 3.1 Intervarietal diversity in starch granule changes induced by HW treatment

**Figure 1:**
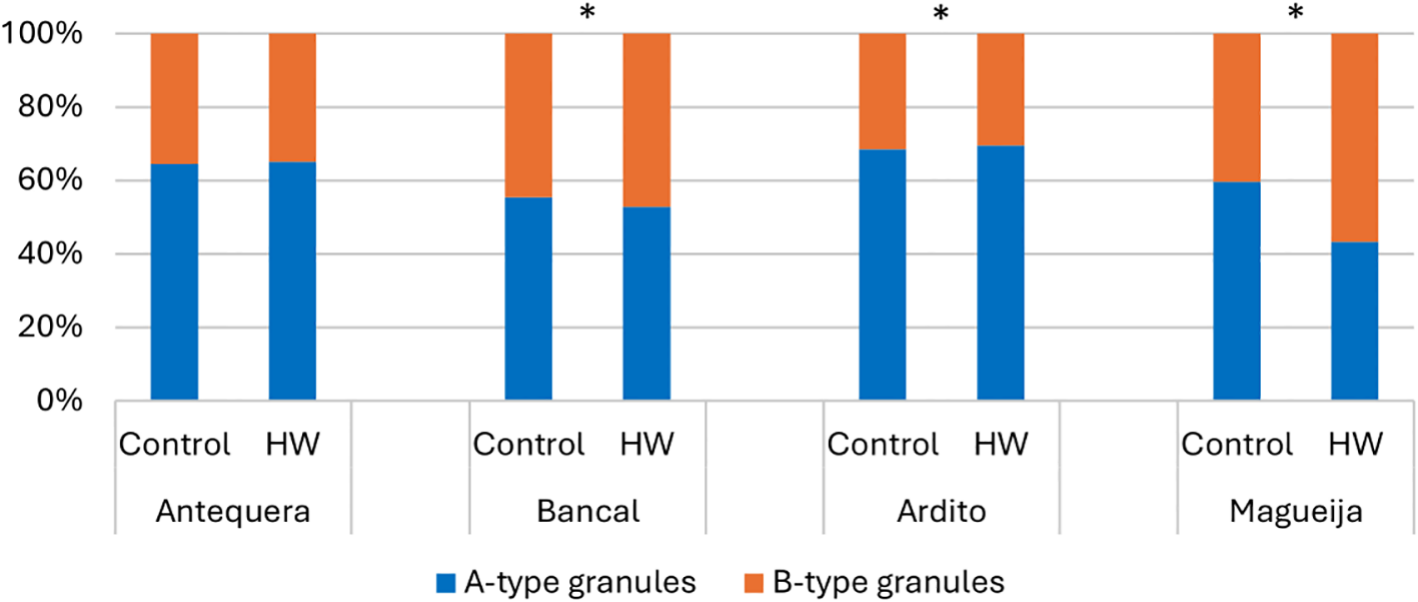
A-type starch granule (blue) and B-type granule (orange) distribution in grains of Antequera and Bancal (commercial varieties) and Ardito and Magueija (Portuguese landraces) obtained from plant submitted to control and heat wave (HW) conditions. *Chi-squared test statistical differences between control and HW in each genotype (p < 0.05).

Since the size distribution of starch granules affects the physicochemical properties of starch and consequently modulates the quality of wheat dough and final product (Guo et al., 2023), the effect of heat waves on the number, dimension, and shape of starch granules was evaluated in grains from control plants and plants subjected to HW treatment (**Supplementary Figure 2**). Granules with a diameter of 10–35 µm were classified as A type, and those with a diameter of <10 µm were classified as B type (Shewry et al., 2020; Brouns et al., 2012). Since it was previously shown that the distribution of starch granules depends on the position of the grain on the wheat spike (Yu et al., 2014), it must be emphasized that all grains analyzed here were collected from the same median region of the first spike of each plant studied. The relative distributions of the different granule types are summarized in **Figure 1** (detailed in **Supplementary Table 1**) and showed significant differences between grains from plants kept in different temperature conditions in all genotypes studied except in the commercial variety Antequera. In Bancal and Magueija, grains from plants subjected to HW treatment showed a reduction of A-type granule number more pronounced in Magueija landrace (16%) than in Bancal (2.67%), and reciprocal B-type granules increase. A reduction in A-type granules has previously been observed in commercial wheat varieties submitted to a daily average temperature of 40°C for 3 days (Liu et al., 2011), as well as in field experiments in late sowing, especially in heat-sensitive lines (Li et al., 2018). However, the opposite was observed in Ardito, where treated plants showed a 1.06% increase in the number of A-type granules and a consequent reduction in the number of B-type granules. Similar increases in the A-type granules were obtained under a 28°C night and 37°C day regime imposed from anthesis to seed maturation, compared to the 17°C night and 24°C day treatment through the evaluation of cv. Butte 86 (Hurkman et al., 2003; Hurkman and Wood, 2011). One of the effects of high temperatures in wheat is the acceleration of grain development with premature grain ripening (Farooq et al., 2011). Consequently, since the initiation and development of B-type starch granules start later in endosperm formation (reviewed in Ma et al., 2018), heat wave-like treatments during the grain-filling stage may induce a relative decrease in B-type granules. However, Liu et al. (2011) observed smaller granules after 1 hour at high temperatures in both susceptible and tolerant genotypes, although the effect was more accentuated in the former. The differential effects of HW-like treatment on the proportion of A- and B-type granules observed in different genotypes also corroborate the report of Rakita et al. (2023), who evaluated eight different cultivars in field experiments.

**Figure 2:**
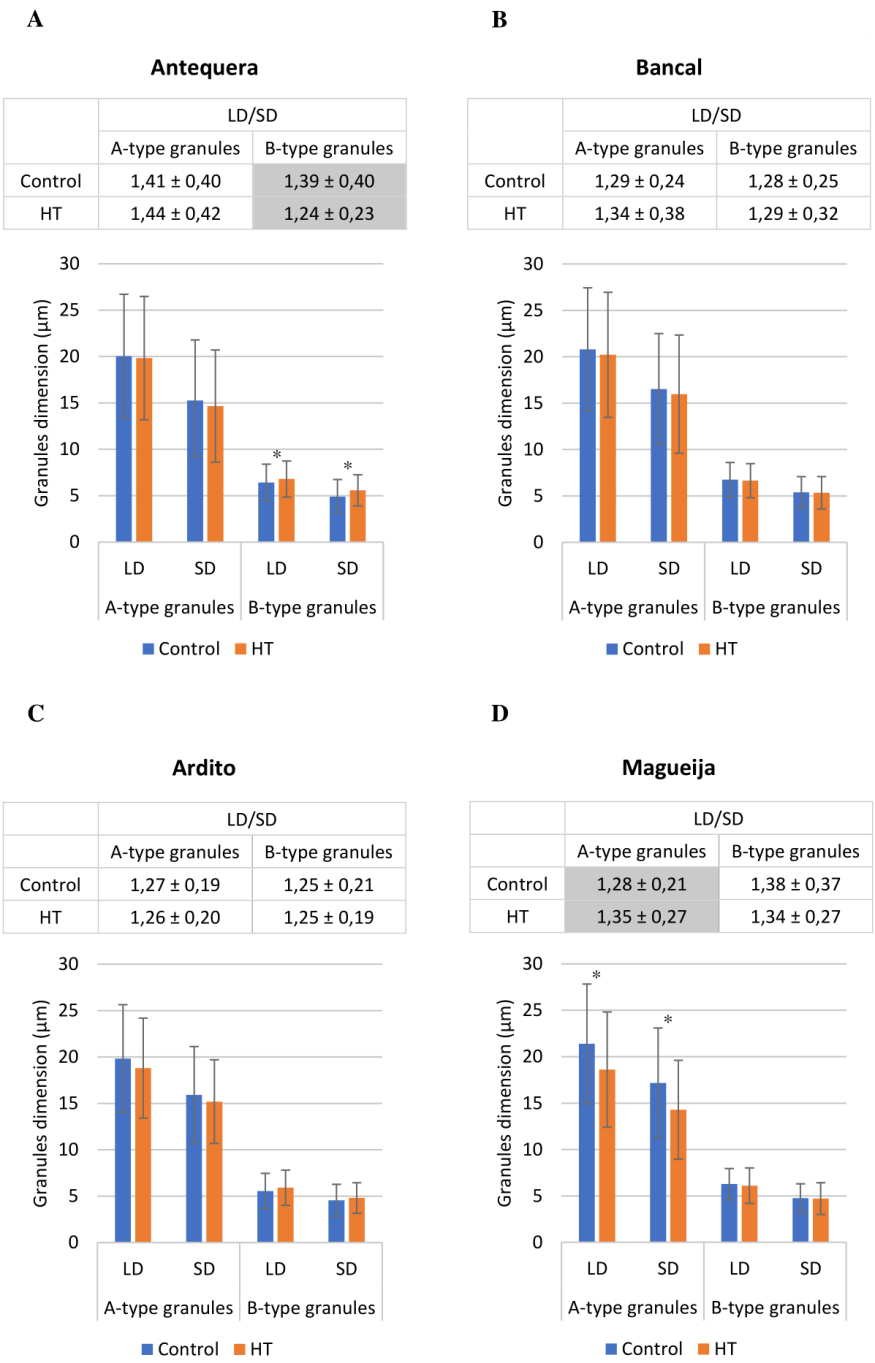
Mean values of starch granule diameter (bottom chart) and diameter ratio (top table) in wheat grains obtained from plants developed in control (blue) and heat wave (HW) conditions (orange) ± standard deviation: **(A)** commercial variety Antequera, **(B)** commercial variety Bancal, **(C)** landrace Ardito, and **(D)** landrace Magueija. LD, largest diameter; SD, smallest diameter; LD/SD, ratio of largest and smallest diameter. *t-Test statistical differences between control and HW in each genotype (p < 0.05). Gray-colored table cells differ significantly in starch granule shape in control and HW (p < 0.05).

To further characterize the effect of HW treatment on starch granules, their largest (LD) and smallest (SD) diameters were quantified, and LD/SD ratios were calculated; the results are presented in **Figure 2** (detailed in **Supplementary Table 2**). The comparison of LD/SD ratios between A-type and B-type granules in each variety/condition confirms that in grains from all genotypes except Magueija control, A-type granules tend to be lenticular, and B-type granules tend to be more spherical, as previously described (reviewed in Shewry et al., 2020). Only in Magueija grains maintained under control conditions the LD/SD ratio of A-type starch granules was significantly lower (1.28) than that of B-type granules (1.38), indicating that in this genotype, the shape of A-type granules is more spherical compared to B-type granules. We can speculate that this finding can be explained by the fact that this genotype is an old landrace collected in the 1930s (Vasconcelos, 1933), which has not been subjected to modern wheat breeding programs, unraveling genetic diversity concerning grain traits related to wheat end-use products. The comparison of granule dimensions in grains from control and HW-treated plants of each genotype, in terms of the LD and SD averages and the ratio between them, showed no significant differences in the commercial variety Bancal and the landrace Ardito, in neither A-type nor B-type starch granules (**Figures 2B, C**, respectively). However, significant differences were disclosed in both the commercial variety Antequera and the landrace Magueija. In Antequera, B-type starch granules showed a significant increase in both the largest (from 6.42 µm to 6.80 µm) and smallest (from 4.89 µm to 5.58 µm) diameter means (**Figure 2A**). Conversely, Magueija A-type granules showed a decrease in both the largest and smallest diameter means (from 21.38 µm to 18.61 µm and from 17.17 µm to 14.28 µm, respectively) (**Figures 2A, D**). Interestingly, in Magueija, the HW treatment inverted the relative shape of the different granule types, as indicated by a significant increase in the LD/SD ratio of A-type granules (**Figure 2D**). In Antequera, an inverse effect was revealed by a significant decrease in the LD/SD ratio, suggesting more spherical B-type granules after HW treatment (**Figure 2A**). Previously, it was reported that starch granules from plants subjected to heat stress above 30°C became smaller and ellipsoidal in shape compared to control environmental conditions (18°C–28°C), an effect that was more pronounced at 40°C (Liu et al., 2011). However, Antequera’s results revealed an opposite effect, as the HW B-type starch granules increased in diameter and acquired a more spherical shape. These results suggest genetic diversity in the response to thermal stress since starch granules’ size and shape are not affected in Bancal and Ardito, but changes in starch granules were induced in grains from Magueija and Antequera. Moreover, the opposite effects of HW treatments reported in starch granules from grains of the two landraces studied highlight the putative increased diversity of this type of germplasm, compared to commercial varieties, in thermal stress response.

### 3.2 Variable effects of heat treatment on endosperm ultrastructure

**Figure 3:**
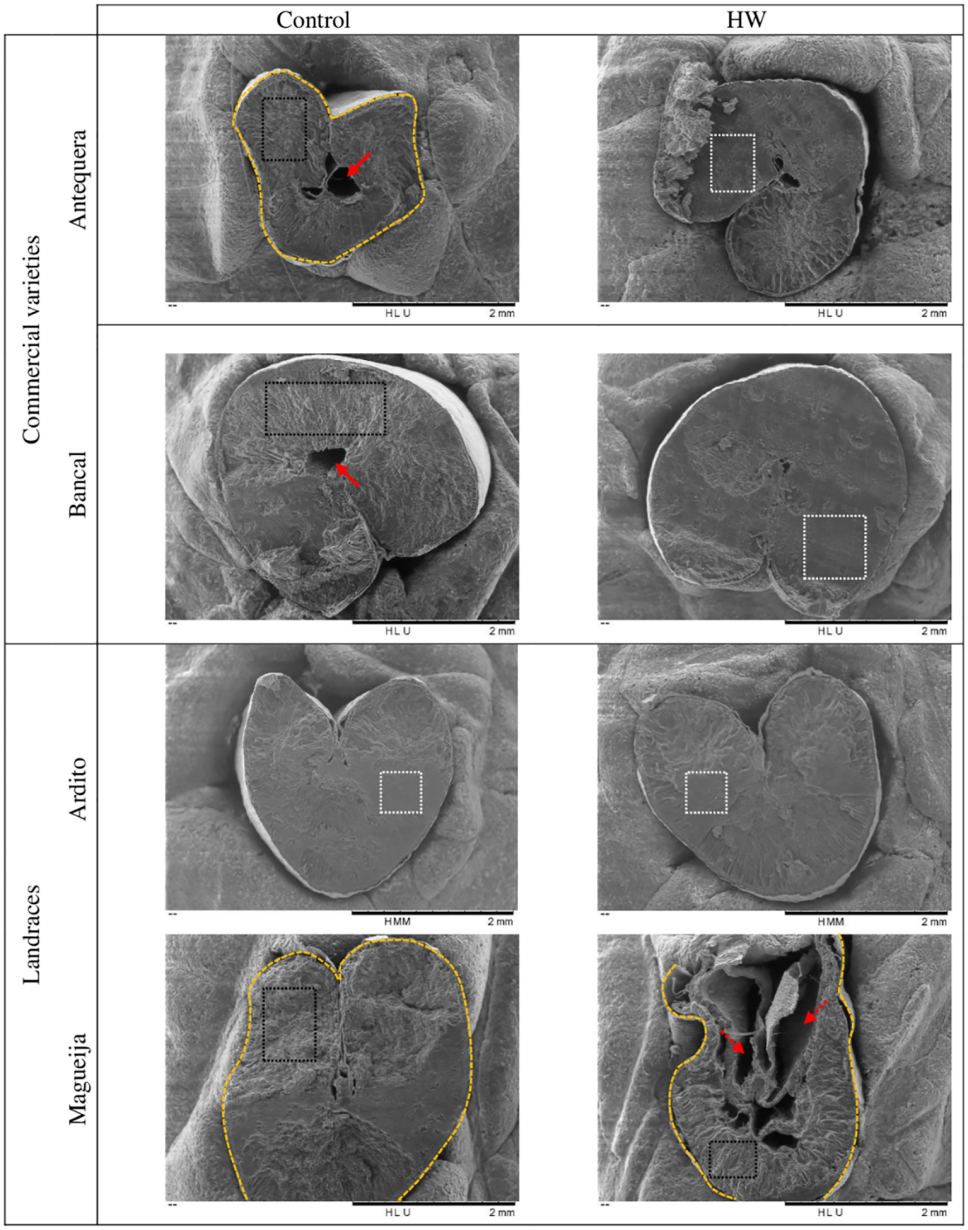
Scanning electron microscopy images of control and heat wave (HW) treated grains of *Triticum aestivum*, representative of the three replicates per each genotype and condition analyzed. Red arrows represent central cavities. Red dashed arrows represent other cavities. Yellow dashed lines represent irregular grain shape. Black dashed rectangles represent uneven areas. White dashed rectangles represent uniform smooth/vitreous areas.

**Figure 4:**
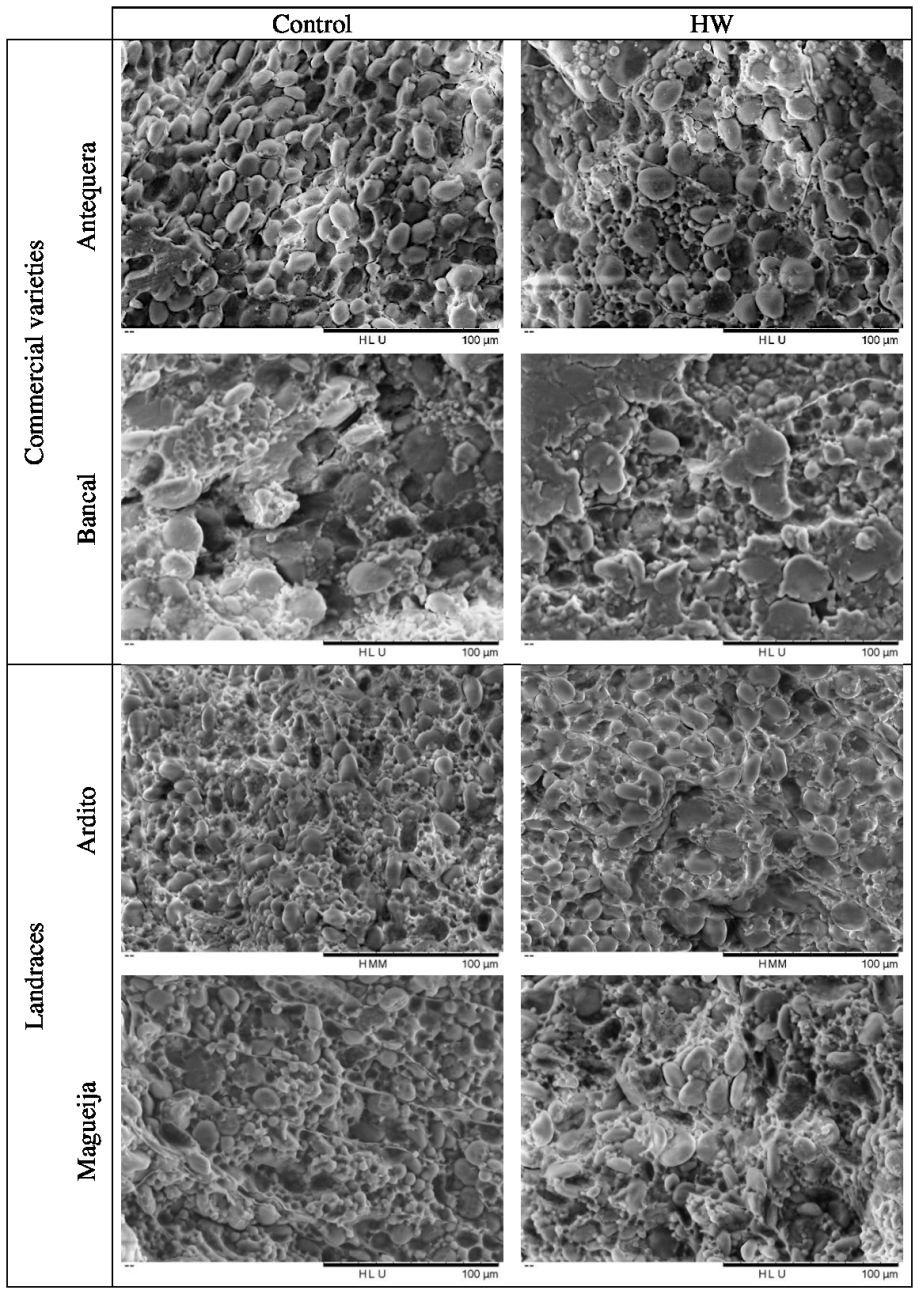
Scanning electron microscopy images of starch granules of control and heat wave (HW) treated grains of *Triticum aestivum*, representative of the three replicates per each genotype and condition analyzed.

Scanning electron microscopy was used to evaluate the morphology and structural aspects of the grains in all the genotypes studied under control and treatment conditions, and representative raw images of the three biological replicates analyzed for each genotype/condition are shown in **Figures 3**, **4**. From a general perspective, it was possible to verify that the grains obtained from both control and treated plants of Antequera are the smallest, a difference that is particularly marked in comparison with Bancal, confirming the significant difference previously detected between these two commercial varieties in terms of ten-grain weight (Tomás et al., 2020b). In addition, the grains of the commercial variety Antequera presented an irregular shape in comparison to those of Bancal, which were the most round-shaped. Other morphological characteristics observed in both commercial varieties Antequera and Bancal were central cavities in the endosperm and uneven smooth/vitreous areas on its surface (**Figures 3A, C**). For the landraces evaluated, no central cavities were detected in the endosperm, but while Ardito grains had a more regular/round shape and a uniform vitreous surface, Magueija grains had an irregular shape and vitreous areas on the endosperm surface (**Figures 3E, G**, respectively).

The comparative evaluation of grains obtained from commercial varieties subjected to HW conditions revealed a more round shape of the endosperm with fewer gaps and an increase in vitreous surface area, changes that were more accentuated in Bancal (**Figures 3B, D**). SEM images of grains from landraces did not reveal major qualitative differences between the control and treated endosperms of Ardito (**Figure 3F**). However, in Magueija, substantial changes were unraveled since grains showed not only a consistently smaller size but also a substantial increase in endosperm cavities and a reduction in vitreous surface (**Figure 3H**), contrary to the effect observed in commercial varieties. The large increase in the number of cavities in Magueija endosperms after HW treatment suggests the occurrence of grain-filling defects under high temperatures. This effect can be explained by insufficient starch synthesis and premature interruption of the grain-filling process previously described using SEM in shriveled wheat grains (Hennen and Brismar, 1987). Additionally, the observed increase in endosperm cavities is in accordance with the significant decrease in ten-grain weight previously observed in landraces under heat stress (Tomás et al., 2020c), although these cavities were not detected in Ardito-treated grains. These consistent changes in grain shrinkage and weight reduction were also observed in other wheat varieties subjected to high-temperature treatments (31°C/20°C) (Dias et al., 2008). Furthermore, Li et al. (2017) detected substantial changes in grain morphology, similar to those detected here in Magueija, induced by elevated temperature (28°C/37°C) imposed from 5 days after anthesis until grain maturity and associated with increased α-amylase activity during grain filling of heat-treated plants, and, as described below, a significant upregulation of α-amylase was also detected in immature grains collected from Magueija plants submitted to HW-like conditions. Additionally, the significant shrinkage induced by HW on Magueija endosperm may also be related to pericarp gene downregulation, associated with limited pericarp cell wall expansion induced by post-anthesis heat previously reported by Kino et al. (2020). These authors suggested that the reduction in mature grain weight may be correlated with gene downregulation induced by high temperature. Endosperm vitreousness is an optical parameter influenced by growth conditions during grain filling and the drying rate at maturity, which is relevant for rheological properties. This morphological parameter of the endosperm may also be related to a higher density of the protein matrix (reviewed in Edwards, 2010). Although vitreousness was observed in all varieties, its increase was more pronounced in Bancal grains obtained under HW conditions. However, Tomás et al. (Tomás et al., 2020b, c) did not find a significant variation in protein content in grains of this commercial variety, suggesting that this variation may depend on other variables.

From the scanning electron microscopy analysis, it was also possible to observe the appearance of the starch granules. In all genotypes and conditions, the starch granules had smooth and wrinkle-free surfaces (**Figure 4**), a specific characteristic of wheat starch granules (Hennen and Brismar, 1987). Although the above analysis of granule distribution showed that the proportion of Antequera granules remained constant under control and treatment conditions (**Figure 1**), SEM images showed a greater number of B-type granules in treated grains (**Figures 4A, B**). However, it should be underlined that the distribution of A-type and B-type granules was assessed in samples from the whole endosperm, whereas the SEM analysis was restricted to a median cross section of each grain. Due to the aforementioned substantial increase of the vitreous surface in Bancal HW- treated grains, it was very difficult to obtain an image distinguishing starch granules compared to the grains kept in control conditions (**Figure 4D**). Similarly, results obtained with higher magnification of Ardito endosperm did not reveal qualitative differences between grains produced in control and treated plants, while in Magueija, the granule disposition was more disorganized in treated grains compared to the control ones (**Figures 4E–H**, respectively).

### 3.3 Transcription evaluation unravels HW impacts on starch synthesis and degradation

There is strong evidence that the response of wheat plants to temperature stress situations results from complex physiological, cellular, and molecular processes with ultimate consequences on the main raw material of wheat (reviewed in Lal et al., 2021). In this context, it is imperative to evaluate the effects of high-temperature treatments mimicking a heat wave, particularly on biological processes with an impact on wheat grain yield and quality. The comparative evaluation of the whole transcriptome of immature filling grains produced by wheat plants grown under control conditions (20°C/25°C) and subjected to a heat wave-like treatment (maximum temperature of 40°C) during grain filling was previously performed (Tomás et al., 2022). That comparison identified a total of 10,366 DEGs, considered significant with an adjusted p-value (padj) <0.05, and up- and downregulated genes were obtained by filtering the log2 fold change absolute value higher than 1, as detailed in Tomás et al. (2022). This previous work reported DEGs assignment of gene ontologies (GOs) for biological processes, molecular functions, and cellular components performed (Tomás et al., 2022), showing that in terms of cellular components, the most represented class was organelle (GO:0043226), encompassing up to more than 50% of DEGs in all four genotypes analyzed, revealing a high impact of heat on cellular functions potentially relevant of starch synthesis. Significant enriched Gene Ontology terms associated with DEGs involved in starch synthesis and degradation, presented in **Supplementary Table S3**, clearly indicate that the genotype most affected by the HW treatment was the landrace Magueija, which may help to understand the marked changes induced in the endosperm of grains from this landrace subjected to this abiotic stress condition revealed by SEM analysis. In fact, Magueija revealed that a higher number of GO terms significantly downregulated, in contrast with the other genotypes studied, and their ontologies most significantly downregulated including categories of carbohydrate biosynthetic process, starch metabolic process, amylopectin metabolic process, and amylopectin biosynthetic process.

The aforementioned comparative analysis of the whole transcriptome was further dissected here regarding differentially expressed genes functionally annotated by KEGG to starch and sucrose metabolism pathways (**Table 1**; complete list and detailed in **Supplementary Table S4**) and specifically regarding genes encoding enzymes associated with starch biosynthesis and degradation (**Table 2**). A primary analysis of these data revealed that none of these genes were differentially expressed in grains from HW-treated plants of the commercial variety Antequera (**Tables 1**, **2**). This genotype was also the most stable with respect to the distribution of A-type and B-type starch granules in mature grains after HW during grain filling, although grains produced by HW-treated plants seemed to have a more uniform shape. Regarding the other genotypes studied, the results summarized in **Table 1** show that more genes related to starch and sucrose metabolism were downregulated (55.4%) than upregulated (44.6%), and the majority of thermostress-induced differentially expressed genes assigned to starch and sucrose metabolism were detected in Bancal and Magueija (46.4% and 48.2%, respectively), and only 5.4% corresponded to Ardito. These results contrast with those reported regarding the whole transcriptome assessment of the same four genotypes reported in Tomás et al. (2022) since in that case, Bancal was the genotype presenting a lower number of DEGs. This contrast is, moreover, evident since the landraces unraveled a considerably higher number of DEGs compared with the commercial varieties—86% of the total DEGs detected were assigned to Ardito and Magueija genotypes, revealing a clear, more responsive transcriptome of old traditional varieties. None of the DEGs encoding enzymes related to starch and sucrose metabolism were common to all genotypes analyzed, as most DEGs common to two genotypes were detected, with no obvious preponderant pattern (**Supplementary Table S4**).

**Table 1:** Differentially expressed genes functional annotated through KEGG to Starch and Sucrose Metabolism pathways in filling grains of commercial varieties Antequera and Bancal and landraces Ardito and Magueija.

	Differentially expressed genes (DEGs)—starch and sucrose metabolism								
	Antequera		Bancal		Ardito		Magueija		Total
**DEG N.**	0	0	30	22	1	5	31	23	112
**Downregulated**	0.0%		26.8%		0.9%		27,7%		55,4%
**Upregulated**		0.0%		19.6%		4,5%		20,5%	44,6%
**Total/variety**	0.0%		46.4%		5.4%		48.2%		

Red and blue indicate down- and upregulated genes, respectively.

KEGG, Kyoto Encyclopedia of Genes and Genomes.

**Table 2:** Differentially expressed genes involved in starch synthesis and degradation in filling grains of commercial varieties Antequera and Bancal and landraces Ardito and Magueija.

Variety	Gene	Encoded enzyme
Glucose-1-phosphate adenylyltransferase (EC 2.7.7.27)		
**Ardito**	TraesCS1A02G419600	AGPase
	TraesCS1B02G449700	
	TraesCS1D02G427400	
**Magueija**	TraesCS1A02G419600	
	TraesCS1B02G449700	
	TraesCS1D02G427400	
Starch synthase (EC 2.4.1.21)		
**Bancal**	TraesCS7D02G190100	SSII
**Ardito**	TraesCS7A02G189000	SSII
	TraesCS7B02G093800	
**Magueija**	TraesCS7A02G120300	SSI
	TraesCS7B02G018600	
	TraesCS7D02G117800	
**Bancal**	TraesCS1A02G091500	SSIII
**Ardito**	TraesCS2A02G468800	GBSSII
	TraesCS2B02G491700	
**Magueija**	TraesCS2D02G468900	GBSSII
	TraesCS2A02G468800	
	TraesCS2D02G468900	
Starch-branching enzymes (EC 2.4.1.18)		
**Ardito**	TraesCS2A02G310300	SBEIIb
	TraesCS7A02G549100	SBEI
**Magueija**	TraesCS2A02G310300	SBEIIb
	TraesCS7B02G472500	SBEI
	TraesCS7D02G535400	SBEI
Alpha-amylase (EC 3.2.1.1)		
**Bancal**	TraesCS2B02G306400	α-Amylase
**Magueija**	TraesCS7A02G383900	
Beta-amylase (EC 3.2.1.2)		
**Ardito**	TraesCS2D02G220900	β-Amylase
**Bancal** **Bancal** **Bancal**	TraesCSU02G032700 TraesCS1B02G229000 TraesCS4D02G006100	
**Magueija**	TraesCS6B02G116400	

Red and blue indicate down- and upregulated genes, respectively.

SS, starch synthase; GBSS, granule-bound starch synthase; SBE, starch-branching enzyme.

Hurkman et al. (2003) revealed by Northern blotting a general decrease in transcripts of all genes encoding enzymes involved in starch synthesis resulting from high temperature imposed from anthesis until seed maturation, studying only one bread wheat variety. However, our results showed that more restricted heat conditions mimicking a heat wave induce more limited effects that vary in different genotypes. In fact, a more specific analysis of DEGs involved in starch synthesis and degradation, presented in **Table 2**, revealed both up- and downregulated genes differentially detected in Ardito, Bancal, and Magueija. However, only one upregulated gene and one downregulated gene encoding for enzymes involved in starch synthesis were detected in Bancal—starch synthase II and starch synthase III, respectively. Thus, starch biosynthesis in commercial cultivars is considerably less affected by heat than in old traditional varieties. These results may be in line with the stability regarding ten-grain weight under HW (Tomás et al., 2020b) and the lack of negative effects on endosperm ultrastructure observed in the commercial varieties studied here. However, one of the 10 more downregulated genes detected in Bancal commercial varieties encodes for a sugar major facilitator transporter (TaSTP10, TraesCS1D02G221100; Tomás et al., 2022). This DEG belongs to the wheat sugar transporter family, which showed variable expression profiles in wheat seedlings under different abiotic stresses (Liu et al., 2020; Prasad et al., 2023) with a decrease in the expression levels after heat stress (Prasad et al., 2023). However, this facilitator transporter family has also been shown to be involved in carbohydrate partitioning and ultimately in rice grain filling (Sun et al., 2022). To our knowledge, this is the first time that changes in wheat sugar transporter expression induced by heat stress have been identified during developing grains.

In contrast to the limited detection of DEGs encoding enzymes required for starch synthesis in commercial varieties, both traditional genotypes studied have a greater and similar response to HW treatment, with a much higher number of DEGs associated with the starch synthesis pathway detected in immature grains, most of which were downregulated. Lu et al. (2019), evaluating the effects of heat stress (22°C/32°C) during grain development in a local Chinese wheat variety, showed also that most genes encoding key enzymes required for starch biosynthesis were downregulated. In the traditional genotypes Ardito and Magueija, three genes encoding glucose-1-phosphate adenylyltransferase (AGPase) are downregulated, implying that HW treatment has an overall detrimental effect on starch synthesis by potentially reducing the number of glucose units since AGPase is a major limiting enzyme in seed starch biosynthesis (Smidansky et al., 2006). Indeed, Rangan et al. (2020) also observed the downregulation of AGPase-encoding genes as a result of heat stress during seed development and associated this trait with heat-susceptible genotypes. Considering starch synthase-encoding genes globally, both landraces revealed the same effects, mainly downregulated genes. Moreover, the results obtained in Ardito and Magueija suggest that the amylose:amylopectin ratio may be increased due to thermostress because SSI, SSII, and SBEI- and SBEII-encoding genes, involved in amylopectin synthesis, are downregulated and GBSSII-encoding genes, required for amylose synthesis, are upregulated. These support the work of Liu et al. (2011), who reported a decrease in starch content due to lower amylopectin synthesis in grains obtained in plants subjected to temperatures above 30°C during 3 days and also with SS downregulation associated with starch content decrease disclosed after 1 hour at 37°C or 40°C (Kumari et al., 2020). In addition, the structure of amylopectin may be different in both genotypes, as SSII is more affected in Ardito, whereas SSI is more affected in Magueija, with putative effects on branch size (reviewed in Wang et al., 2014).

Regarding the enzymes involved in starch breakdown, a much greater number of genes encoding amylase, both α- and β-amylase, were downregulated in the Bancal commercial variety compared to the other genotypes studied. These differences induced by HW on the transcription of amylase genes in filling grains, which affect starch dynamics, may have reverberated in mature grains since in this variety more round-shaped endosperm with fewer gaps was observed by SEM, suggesting a more efficient grain filling. Regarding the old traditional varieties studied, in Ardito, only one gene encoding β-amylase was upregulated; in Magueija, one gene encoding β-amylase was downregulated, and one gene encoding α-amylase was upregulated after HW-like stress. β-Amylase enzymes (EC 3.2.1.2) release β-maltose from polyglucans produced during grain filling, which are major proteins of wheat starchy endosperm (Zhang et al., 2023b). β-Amylase has been much less studied than α-amylase, and to our knowledge, this is the first time that heat-induced modulation of the expression of genes encoding these starch-degrading enzymes has been reported. The relevance of α-amylase upregulation for grain development, which may ultimately affect wheat yield, has been previously reported and is consistent with the association of α-amylase upregulation and a substantial alteration in endosperm morphology that was disclosed in Magueija, specifically with a large increase in the occurrence of endosperm cavities. In fact, α-amylase function during seed germination is obvious, while its activity may be detrimental during grain development, assuming late maturity α-amylase increasing relevance in wheat breeding (reviewed in Zhang et al., 2022). This prematurely produced α-amylase may be conserved throughout grain maturation, resulting in a low falling number and quality decline and may depend on genotype and abiotic stresses like temperature (Derkx and Mares, 2020). This concern is aggravated by the increase in α-amylase associated with grain size reduction detected in a broad panel of wheat landraces for wide geographical distributions including those in Portugal subjected to a 10-day treatment with a maximum daily temperature of 36°C imposed 20 DAA (Barrero et al., 2020). Paul et al. (2022) found heat-responsive genes encoding enzymes involved in several molecular processes, including also starch synthesis pathway during grain filling, highlighting among them an α-amylase inhibitor that protects starchy grain reserves from degradation. Upregulation of this gene has been suggested to be involved in heat stress tolerance (Paul et al., 2022), but in the genotypes assessed here, this gene was not differentially expressed in none of the genotypes studied here. The upregulation of the α-amylase gene disclosed in Magueija together with the downregulation observed in genes encoding enzymes involved in amylopectin synthesis (both SS and SBE), which constitutes the majority of the starch composition (~ 70% to 80%) (Shewry, 2009), may be responsible for the marked endosperm alteration revealed by SEM in this old traditional variety, which is reflected in a reduction of the vitreous surface, grain size, and substantial increase in irregular endosperm gaps. Conversely, the absence of adverse effects of HW stress observed in Antequera regarding starch granule distribution, endosperm morphology, and DEGs involved in starch and sucrose metabolism pathways, particularly in starch synthesis and degradation, point to this variety as a potentially stable one to be used in global warming scenarios.

Globally, our results suggest that starch amount and composition can be strongly affected, mainly in traditional genotypes, and these results can be contextualized by broader effects deciphered in Tomás et al. (2022), showing that within the pathways correlated with downregulated genes, carbohydrate metabolism was the most affected in Bancal and both traditional genotypes. The overall variable consequences induced by heat stress in wheat landraces contrastingly with the more similar response observed in commercial varieties highlights the potential usefulness of the biodiversity contained in old traditional wheat genotypes facing climate change. Moreover, the discrepancy of molecular adjustments between commercial and traditional genotypes in pathways involved in heat response contributes to understanding the relevance of genetic diversity to cope with these extreme events. In the future, we intend to extend the present study through NMR, which is used to monitor the structural and functional starch granules (Wang et al., 2024) related to physicochemical properties important for wheat end-use products, to further study changes induced by HW. This analysis will also allow a deeper interpretation of the SEM endosperm/granule assessment performed in this study. Moreover, the present study was limited to the starch fraction of wheat grain, which is its major compound with crucial nutritional and technological implications. However, in the future, we intend to additionally evaluate HW treatments’ impact on other wheat grain compounds with food value implications. We are especially interested in the assessment of food contaminant modulation by heat during grain filling, namely, in acrylamide that is produced from free asparagine during high-temperature processing. In fact, the effect of abiotic stress on free asparagine levels continues to be a relevant scientific topic (Oddy et al., 2023), and we are particularly interested in the exploitation of wheat intraspecific variability regarding this issue. Finally, this work addresses the effects of a single abiotic stress condition under strictly controlled conditions, but it will ultimately be mandatory to assess complex situations with different simultaneous stress conditions simulating what occurs in wheat fields.

## 4 Conclusion

This study investigated the effects of HWs imposed during grain filling on starch granules, endosperm ultrastructure, and gene expression in different bread wheat genotypes. Interestingly, our results revealed intervarietal diversity in the HW response, highlighting the potential of genetic resources contained in old traditional varieties to diminish the adverse effects of climate change. HW treatments induced changes in endosperm ultrastructure, starch granule distribution, size, and shape, especially in the landrace Magueija. Whole transcriptome analysis revealed a complex HW response affecting the expression patterns of genes involved in starch synthesis and degradation. Overall, traditional landraces showed a more pronounced transcriptional response to HW than commercial varieties, suggesting their potential for adaptation to changing environments. The results obtained in this work emphasize the importance of screening wheat genetic diversity to identify genotypes with enhanced resilience to abiotic stress, suggesting also that further investigations are needed to explore the underlying molecular mechanisms of wheat plasticity under abiotic stress.

## Data Availability

All RNA Sequencing data is available in Sequence Read Archive (SRA), with the project ID PRJNA750265 (https://www.ncbi.nlm.nih.gov/bioproject/750265) under the accessions SAMN20447565, SAMN20447566, SAMN20447567, SAMN20447568, SAMN20447569, SAMN20447570, SAMN20447571, SAMN20447572.
