# Supplementary material for: Deciphering heat wave effects on wheat grain: focusing on the starch fraction
Source: Front Plant Sci. 2024 Dec 4;15:1459283. doi: 10.3389/fpls.2024.1459283 (PMC11659670; doi:10.3389/fpls.2024.1459283)
Supplement: Supplementary file 1 [file DataSheet1.zip › Data Sheet 1/Supplementary Material Tables 1, 2 and Figure 1, 2.docx]

Supplementary Material

# Supplementary Tables

**Supplementary Table 1.** Distribution of A- and B-type starch granules of Ardito and Magueija (Portuguese landraces) and Antequera and Bancal (commercial varieties) obtained from plant developed in control and HW conditions.

|  | | A-type granules | | B-type granules | | Total of  granules  observed |
| --- | --- | --- | --- | --- | --- | --- |
|  |  | Number | Percentage | Number | Percentage |  |
| Antequera | Control | 275 | 64.55 | 151 | 35.45 | 426 |
|  | HW | 276 | 65.09 | 148 | 34.91 | 424 |
|  | Chi-sq test | 0.8 |  | | | |
| Bancal | Control | 178 | 55.45 | 143 | 44.55 | 321 |
|  | HW | 133 | 52.78 | 119 | 47.22 | 252 |
|  | Chi-sq test | 7.48E-06 |  | | | |
| Ardito | Control | 217 | 68.45 | 100 | 31.55 | 317 |
|  | HW | 269 | 69.51 | 118 | 30.49 | 387 |
|  | Chi-sq test | 3.47E-04 |  | | | |
| Magueija | Control | 174 | 59.59 | 118 | 40.41 | 292 |
|  | HW | 164 | 43.27 | 215 | 56.73 | 379 |
|  | Chi-sq test | 2.71E-11 |  | | | |

**Supplementary Table 2.** Mean values of starch granules dimensions in grains from Ardito and Magueija (Portuguese landraces) and Antequera and Bancal (commercial varieties) obtained from plant developed in control and heat wave (HW) conditions.

|  |  | A-type granules  mean values | | B-type granules  mean values | | Largest diameter/Smallest diameter mean values | | Total of  granules  observed |
| --- | --- | --- | --- | --- | --- | --- | --- | --- |
|  |  | Largest  diameter  (µm) | Smallest  diameter  (µm) | Largest  diameter  (µm) | Smallest  diameter  (µm) | A-type granules | B-type granules |  |
| Antequera | Control | 20.03  ±6.68 | 15.25  ±6.54 | 6.42  ±1.98 | 4.89  ±1.86 | 1.41  ±0.40 | 1.39  ±0.40 | 426 |
|  | HW | 19.83  ±6.65 | 14.66  ±6.05 | 6.80  ±1.94 | 5.58  ±1.68 | 1.44  ±0.42 | 1.24  ±0.23 | 424 |
|  | t-test | 0.73 | 0.27 | 9.83E-02 | 9.63E-04 | 0.38 | 6.27E-05 |  |
| Bancal | Control | 20.80  ±6.63 | 16.52  ±5.98 | 6.75  ±1.85 | 5.39  ±1.69 | 1.29  ±0.24 | 1.28  ±0.25 | 321 |
|  | HW | 20.21  ±6.74 | 15.97  ±6.37 | 6.64  ±1.84 | 5.34  ±1.74 | 1.34  ±0.38 | 1.29  ±0.32 | 252 |
|  | t-test | 0.44 | 0.44 | 0.64 | 0.84 | 0.21 | 0.99 |  |
| Ardito | Control | 19.83  ±5.81 | 15.90  ±5.23 | 5.56  ±1.90 | 4.56  ±1.72 | 1.27  ±0.19 | 1.25  ±0.21 | 317 |
|  | HW | 18.80  ±5.39 | 15.19  ±4.51 | 5.91  ±1.90 | 4.80  ±1.65 | 1.26  ±0.20 | 1.25  ±0.19 | 387 |
|  | t-test | 0.05 | 0.11 | 0.17 | 0.30 | 0.34 | 0.94 |  |
| Magueija | Control | 21.38  ±6.45 | 17.17  ±5.92 | 6.27  ±1.68 | 4.75  ±1.55 | 1.28  ±0.21 | 1.38  ±0.37 | 292 |
|  | HW | 18.61  ±6.21 | 14.28  ±5.33 | 6.10  ±1.91 | 4.71  ±1.71 | 1.35  ±0.27 | 1.34  ±0.27 | 379 |
|  | t-test | 7.40E-05 | 3.93E-06 | 0.42 | 0.84 | 0.01 | 0.25 |  |

**Note:** Values are expressed as means ± standard deviation. Grey coloured t-tests differ significantly in the means of starch granules dimensions between control and HW (p < 0.05).

# Supplementary Figures

#
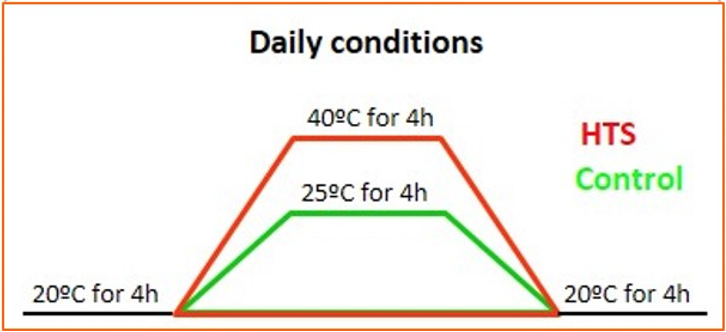


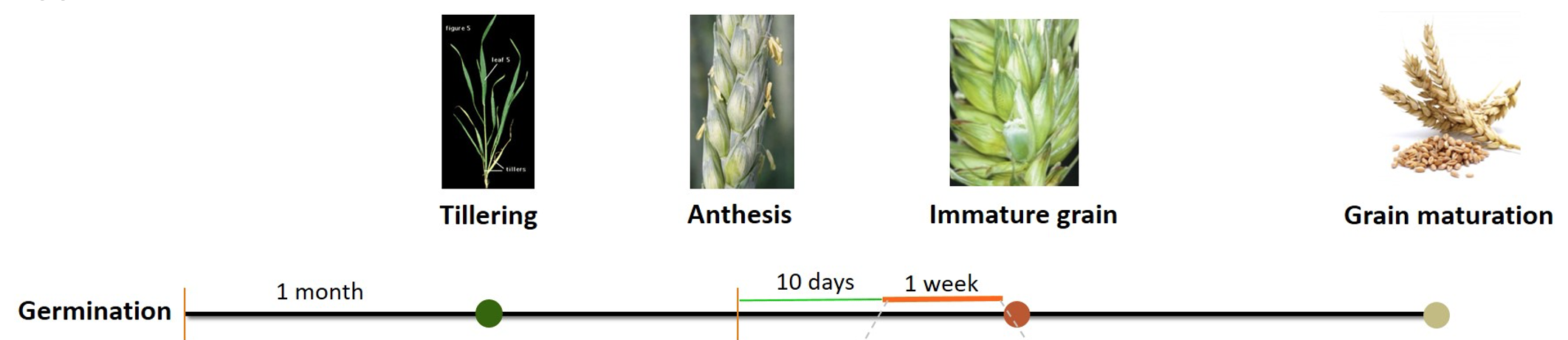

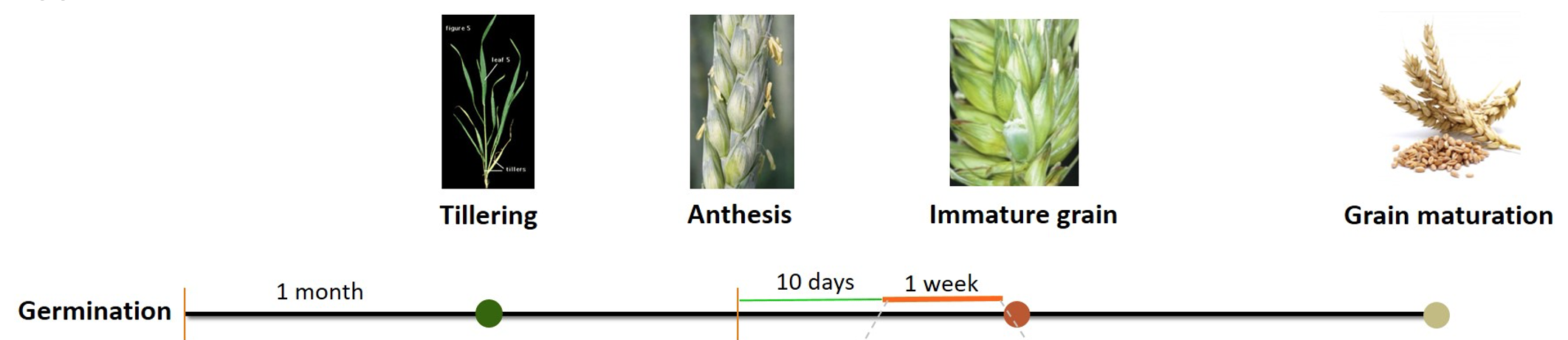


RNA Sequencing

Starch granules and endosperm analysis

**Supplementary Figure 1.** Schematic representation of plant growth conditions and material collection points. Ten days after anthesis, one week treatment mimetizing a heat wave (HW) was imposed in growth chambers with controlled conditions at 8h dark / 16h light cycle. During the 16 h light, temperature progressive increased from 20 ºC to 40 ºC (20 ºC to 25 ºC in control), was maintained during 4h, and then progressive decreased back to 20 ºC. Immature grains were collected at the end of the week treatment for RNA sequencing and mature grains were collected at the end of life cycle for starch granules and endosperm analysis.


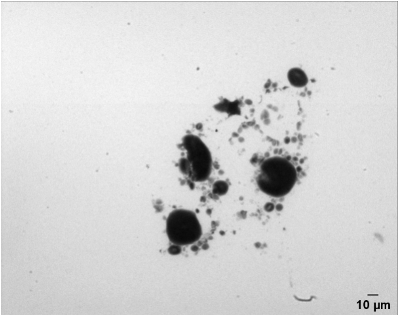


**B**


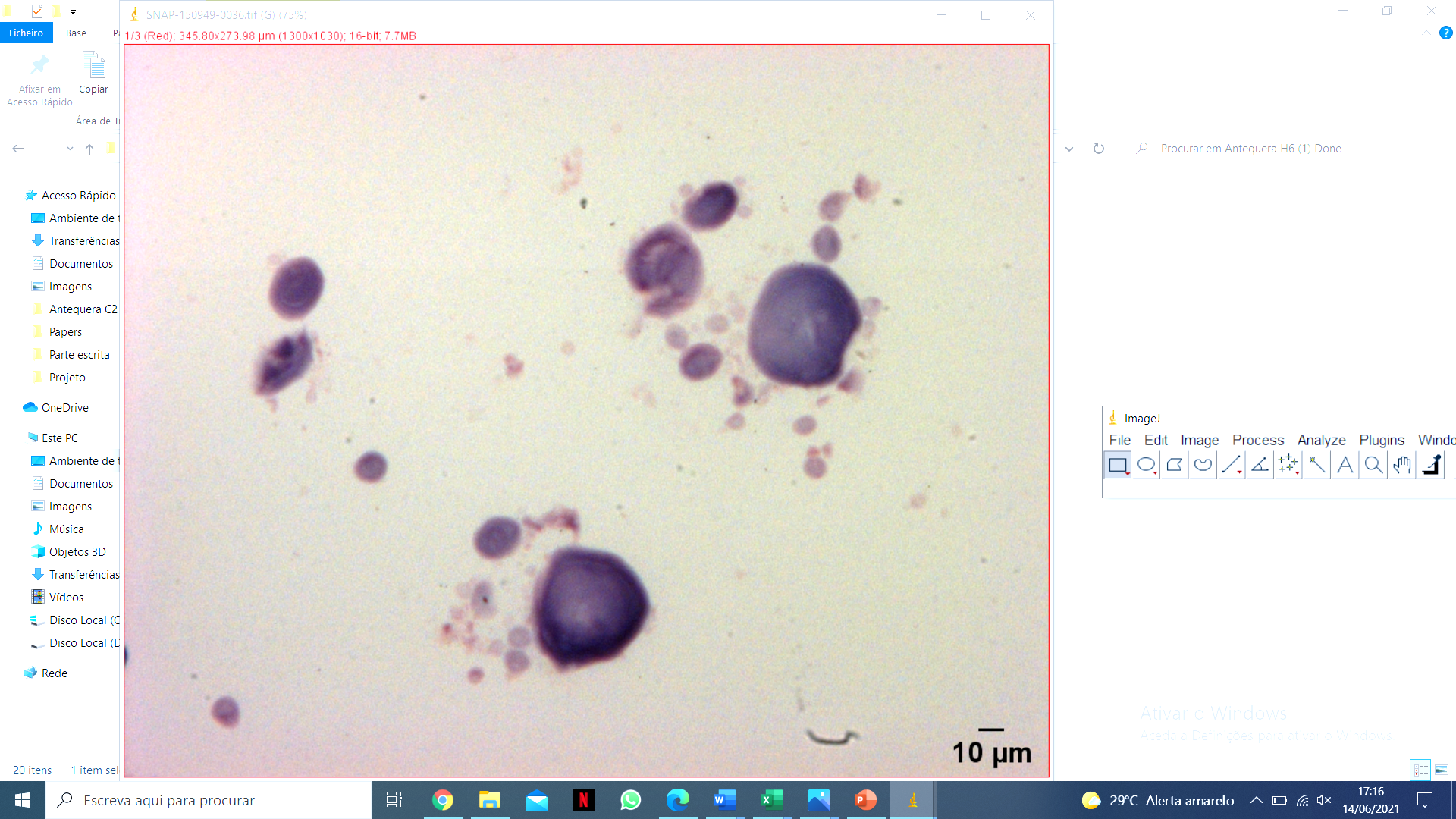

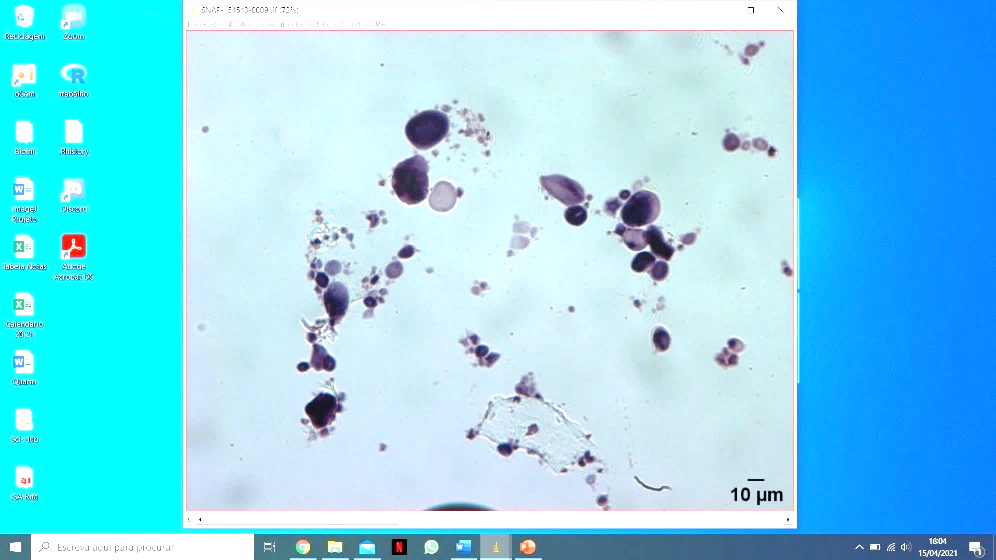


**A**

**D**


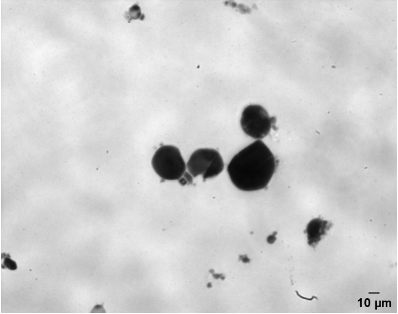


**C**

**Supplementary Figure 2.** Lugol-stained starch granules observed in wheat flour from the commercial variety Antequera (A – control, B – HW) and the landrace Magueija (C – control, D – HW). Bars represent 10 µm.
